# Effects of Smoking on Neurocognitive Outcomes in Patients with Carbon Monoxide Poisoning

**DOI:** 10.3390/jcm14072497

**Published:** 2025-04-06

**Authors:** Eunsan Ko, Yeonjae Park, Yong Sung Cha, Je Seop Lee, Sun Chul Lee, Gyo Jin Ahn

**Affiliations:** 1 Department of Emergency Medicine, Yonsei University Wonju College of Medicine, Wonju 26426, Republic of Koreaemyscha@yonsei.ac.kr (Y.S.C.); 2 Department of Biostatistics and Center of Biomedical Data Science, Yonsei University Wonju College of Medicine, Wonju 26426, Republic of Korea; aceail@yonsei.ac.kr; 3 Research Institute of Hyperbaric Medicine and Science, Yonsei University Wonju College of Medicine, Wonju 26426, Republic of Korea; 4 Department of Emergency Medicine, Inha University College of Medicine, Incheon 22212, Republic of Korea; swf365@naver.com; 5 Department of Physiology, Yonsei University Wonju College of Medicine, Wonju 26426, Republic of Korea

**Keywords:** carbon monoxide, cohort study, poisoning, prognosis, smoking

## Abstract

**Background/Objectives:** The concept of the “smoker’s paradox” in prior research posits that smoking could potentially offer neuroprotective effects in cases of acute carbon monoxide (CO) poisoning. This study aimed to determine the validity of this hypothesis by minimizing selection bias and confounding variables in a comparison of neurocognitive outcomes between smokers and non-smokers following acute CO poisoning. **Methods:** A total of 1150 patients were included in this retrospective study. Propensity Score Matching (PSM) was used to control for variables such as age, initial Glasgow Coma Scale (GCS) score, co-morbidities, and HBO_2_ therapy application. Neurocognitive outcomes were assessed and compared between smokers and non-smokers. **Results:** In the initial analysis, 1150 patients were divided into non-smoking (61.7%) and smoking (38.3%) groups. Before PSM, smokers had a lower rate of poor outcomes (12.1% vs. 6.8%, *p =* 0.004). However, baseline differences emerged, with non-smokers being older with longer CO exposure, while smokers had more males and higher rates of intentional CO poisoning and drug ingestion. Smokers had a 0.56 times lower relative risk of poor outcomes compared to non-smokers (95% CI: 0.38–0.84, *p =* 0.004). After meticulous 1:1 PSM with 15 covariates, 317 patient pairs were matched, creating balanced cohorts. Neurocognitive outcomes at 1 month post-CO exposure showed no significant differences between matched non-smoking and smoking groups (9.8% vs. 7.9%, *p* = 0.461). Post-PSM, the relative risk for poor outcomes remained approximately 0.81 times lower in smokers compared to non-smokers, but with no statistical significance [0.81 (95% CI: 0.49–1.33, *p =* 0.402)]. **Conclusions:** Our findings do not support the idea of a protective effect from smoking in the context of acute CO poisoning. After accounting for potential confounders through PSM, we found that smoking status was not significantly associated with more favorable neurocognitive outcomes.

## 1. Introduction

In the United States, more than 100,000 patients visit emergency department(ED) with CO poisoning every year, and approximately 1000 to 1300 deaths occur due to carbon monoxide poisoning [1,2]. Beyond direct life-threatening risks, CO poisoning has an influence on the cardiovascular [3], renal [4], and neurological systems [5]. Neurocognitive sequelae are one of the most important problems in patients with CO poisoning. Some patients with CO poisoning experience neurocognitive sequelae, including amnesia, parkinsonism, mutism, aphasia, fecal and urinary incontinence, gait disturbances, and coma. Some of these occur after a latency period of 2 to 40 days and can persist permanently, causing greater distress to patients and caregivers [6,7,8]. These factors make it crucial to predict which patients will experience neurocognitive sequelae in order to plan treatment and make follow-up decisions. Numerous clinical and laboratory metrics have been suggested as dependable markers for predicting adverse neurocognitive outcomes in CO poisoning [6,7,9,10,11,12,13,14,15,16,17,18]. These include age, any interval loss of consciousness, Glasgow Coma Scale (GCS) score, serum creatine kinase, longer CO exposure intervals, corrected QT interval prolongation, serum lactate, abnormal findings on diffusion-weighted magnetic resonance imaging.

Although smoking is a well-documented risk factor for various diseases, including stroke and myocardial infarction [19,20], some studies suggest that smoking status appears to have a protective effect on the prognosis of these diseases [21,22,23]. Like in cardio-cerebrovascular conditions, hypoxia is one of the main mechanisms of injury in CO poisoning. The transcription factor hypoxia-inducible factor (HIF)-1, when stabilized by hypoxia, regulates numerous genes, including glycolytic enzymes, enzymes that reduce basal respiratory rate, and vascular endothelial growth factor (VEGF), which are essential for survival in hypoxia [24]. Cigarette smoke elevates HIF-1 activity in cells under normoxic partial pressure [25]. Based on this information, it appears that the prognosis of CO poisoning may be influenced by smoking history. The relationship between smoking and CO poisoning, however, have not been thoroughly studied. Therefore, we aimed to determine whether smoking status could predict neurocognitive outcomes one month following acute CO exposure.

## 2. Materials and Methods

### 2.1. Study Design and Setting

The dataset used in this study was obtained from a cohort at a single tertiary academic hospital. The ED is located in a suburban, tertiary-care hospital and is designated as a regional emergency medical center, which has >46,000 annual patient visits, with 34 beds in the ED. Our hospital established a prospective registry for CO poisoning patients beginning in January 2006. Patient data were retrospectively retrieved from this registry for cases occurring between January 2006 and July 2020. Subsequently, from August 2020 onward, data collection continued prospectively as part of the CARE CO cohort study (ClinicalTrials.gov identifier: NCT04490317), following informed consent. This study utilized data collected from January 2006 through July 2022. This study received ethical approval from the Institutional Review Board of our hospital (IRB number: CR323031) and was conducted in accordance with the Declaration of Helsinki. All patient data were anonymized prior to analysis.

We included patients with acute CO poisoning who visited the ED. In our institution, acute CO poisoning was clinically diagnosed by considering the following factors: (1) clinical presentation indicative of CO poisoning, (2) recent history of CO exposure, (3) COHb concentration greater than 5% for non-smokers and greater than 10% for smokers [5]. Exclusion criteria for this study were as follows: (1) individuals below the age of 19, (2) a documented history of previous CO poisoning, (3) a pre-existing history of neurocognitive dysfunction or cerebrovascular diseases, including dementia, stroke, or Parkinson’s disease prior to the CO poisoning event, (4) the presence of severe comorbid conditions, such as advanced-stage cancer, (5) administration of specific adjunctive treatments, including therapeutic hypothermia or corticosteroids, (6) incomplete data for pivotal variables, including smoking history, and (7) failure to follow up for neurocognitive status after discharge.

CO poisoning patients underwent treatment involving the delivery of 100% high-flow oxygen via a face mask with a reservoir bag. Patients with any interval of loss of consciousness, neurocognitive symptoms or signs, cardiovascular dysfunction, elevated cardiac enzymes, ischemic electrocardiogram changes, severe acidosis, or CO-Hb ≥ 25% were treated with hyperbaric oxygen therapy (HBO_2_) [26]. This was administered within either multiplace or monoplace hyperbaric chambers (IBEX Medical Systems, Seoul, Republic of Korea). The initial hyperbaric session involved a compression to 2.8 or 3.0 atmospheres absolute (ATA) for 45 min, followed by a 60 min treatment at 2 ATA. If a subsequent hyperbaric session was feasible within a 24 h window, a 90 min treatment at 2 ATA was administered.

### 2.2. Study Variables and Definitions

Clinical variables assessed in patients with CO poisoning included the following: age, gender, intentionality of CO poisoning, origin of CO exposure (charcoal, gas and oil, or fire), drug co-ingestion, initial Glasgow Coma Scale (GCS) score at the time of rescue or ED presentation, comorbidities (hypertension, diabetes mellitus, cardio-vascular diseases, and previous psychiatric illness), alcohol co-ingestion, current smoking status, symptoms or signs (loss of consciousness, seizure, or shock), use of HBO_2_, number of HBO_2_ within 24 h, and maximal CO exposure time. In order to categorize the patients’ smoking status, we determined whether they were smokers at the time of the CO poisoning. Information was first gathered from the patient or legal guardian during the ED visit. If information was not acquired initially, it was verified again either after hospitalization or at the 1-month follow-up. Patients for whom data could not be collected in spite of these efforts were excluded in the study due to insufficient data collection. Shock was defined when vasopressor was needed and lactate level was greater than 2 mmol/L [27]. CO exposure duration was estimated by the patients’ guardians as the maximum time interval between the onset of normal consciousness and the detection of the patient. Collected laboratory data included serum CO-Hb, bicarbonate, creatinine, lactate, creatine kinase, and troponin I, all of which were measured within one hour of ED arrival.

The neurocognitive outcomes related to CO exposure were evaluated at one month after discharge through visits to the rehabilitation outpatient department, utilizing the Global Deterioration Scale (GDS) score. The score ranges from 1 to 7, with higher scores representing more severe impairment (See Table S1 for further details) [28]. This scale is also utilized in prognostic evaluations of CO poisoning cases [3,29,30]. In instances where patients in critical condition were unable to attend in person, interviews were conducted with their caregivers. GDS scores were categorized into favorable (1–3) and poor (4–7) neurocognitive outcomes [3,29,30]. Patients who died within 1 month due to CO poisoning (CO-related deaths) were assigned a GDS score of 7. Those who died from unrelated causes were classified as having missing GDS data and were, thus, excluded from the analysis. To further assess longer-term outcomes, we also conducted an additional analysis of neurocognitive status at 6 months post exposure, using the same cohort and statistical methodology as in the primary analysis.

### 2.3. Statistical Analysis

The data were analyzed using various statistical methods to compare variable characteristics. Categorical data were reported as frequencies and percentages, and comparisons were performed with Pearson’s chi-square tests. To assess the normality of continuous variables, we used the Shapiro–Wilk test. Continuous data were presented as median (interquartile range) and were compared using Mann–Whitney U tests. After performing propensity score matching (PSM) procedures, we compared the baseline covariates between the groups (smoker and non-smoker groups). Wilcoxon’s signed-rank tests were used to compare continuous variables, while McNemar’s tests were used to compare categorical variables.

To minimize the potential impact of selection bias and confounding variables, we employed PSM methodology in our study. Specifically, we matched participants based on the following variables: age, sex, intentionality, GCS score, history of diabetes and hypertension, loss of consciousness, presence of shock, use of hyperbaric oxygen therapy, number of hyperbaric oxygen therapy sessions, duration of carbon monoxide exposure (in hours), bicarbonate levels (in mmol/L), lactate levels (in mmol/L), creatine kinase levels (in U/L), and troponin I levels (in ng/mL). These variables are known as common factors influencing patient outcomes or factors previously associated with the prognosis of CO poisoning patients [9,10,14,15,16,17,18,31,32,33,34,35,36]. By conducting this matching, we aimed to evaluate only the impact of smoking status on outcomes. We conducted 1:1 propensity score matching using a greedy matching algorithm, ensuring no replacement of matched pairs, employing a caliper width of 0.25 times the standard deviation (SD) of the logit of the propensity score to achieve balanced groups. [37,38].

The data were analyzed using a chi-squared test and Relative Risk (RR) to examine the relationship between smoking status and outcome status before and after matching. The chi-squared test was used to compare the observed and expected frequencies of outcome occurrence in the smoker and non-smoker groups before and after matching. The Relative Risk was calculated to measure the association between smoking status and poor outcome occurrence before and after matching.

All reported *p*-values were two-sided, with values less than 0.05 regarded as statistically significant. Statistical analyses were performed with SAS 9.4 (SAS Institute Inc., Cary, NC, USA) and R 3.6.3 (R Foundation for Statistical Computing, Vienna, Austria).

## 3. Results

### 3.1. Characteristics of the Study Population

**Figure 1:**
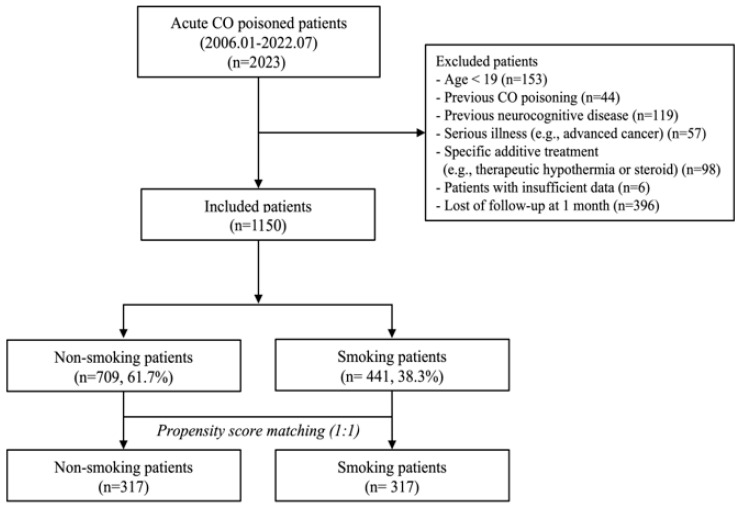
A flow chart of patient selection. CO: carbon monoxide.

After applying the inclusion and exclusion criteria, 1150 patients were included in the final analysis (Figure 1). Based on the 1-month GDS scores after CO poisoning, patients were classified into two group: non-smoking group (709 patients, 61.7%) and smoking group (441 patients, 38.3%). In the unmatched cohort, Table 1 displays the baseline characteristics of the included patients categorized by their smoking status. Patients who did not smoke were older than patients in the smoking group (50 years vs. 41 years, *p <* 0.001). In the non-smoking group, there was a higher proportion of females (*p <* 0.001). Intentional CO poisoning and concurrent drug ingestion were more prevalent in the smoking group (*p <* 0.001 and *p =* 0.014, respectively). The non-smoking group had more patients with hypertension (*p <* 0.001) and fewer patients with psychiatric disease (*p <* 0.001) than the smoking group. The smoking group had more patients with alcohol co-ingestion than the non-smoking group (*p <* 0.001). Patients in the smoking group were more likely to have experienced loss of consciousness (*p =* 0.025). Non-smoking group patients had longer CO exposure times (*p =* 0.007) than the smoking group. In terms of laboratory parameter results, patients of the smoking group showed higher levels of serum lactate (*p =* 0.014), creatinine (*p <* 0.001), and creatine kinase (*p =* 0.027) than those of the non-smoking group. There was a statistically significant difference in neurocognitive outcomes at one month between the two patient groups categorized based on smoking status. Among the patients in the non-smoking group, 86 individuals (86 of 709, 12.13%) exhibited poor outcomes, which was higher than the poor outcome rate among patients in the smoking group (30 of 441, 6.8%) (*p =* 0.004).

### 3.2. Characteristics of Patients After Propensity Score Matching

**Figure 2:**
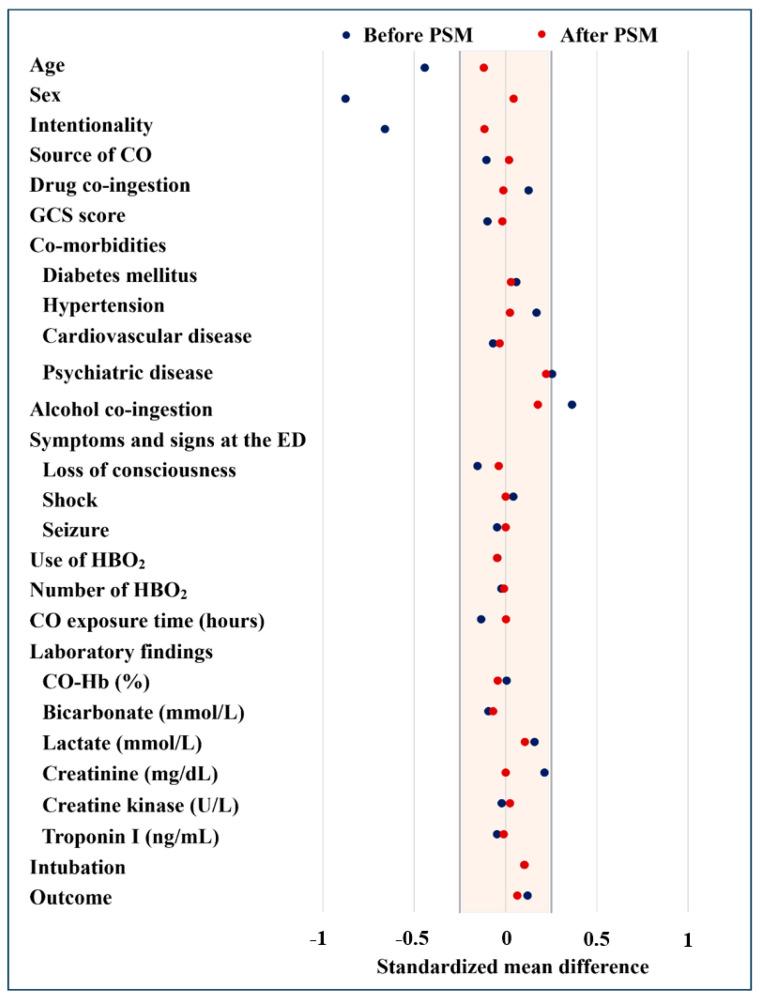
Balance of covariates between the groups. PSM: propensity score matching, CO: carbon monoxide, GCS: Glasgow coma scale, ED: emergency department, HBO_2_: hyperbaric oxygen, CO-Hb: carboxyhemoglobin.

Using 1:1 propensity score matching with 15 covariates across the entire study population, we identified 317 matched patient pairs. The resulting matched cohorts exhibited no significant differences between the non-smoking and smoking groups, except for the rate of patients with a history of psychiatric disease and alcohol co-ingestion (Table 1). To ensure the validity of our study, we evaluated the balance of covariates between the matched groups. The standardized mean differences for the critical 15 covariates used in the PSM analysis were all below 0.25 (Table 1). Consequently, we can confirm that the matched groups displayed a well-balanced distribution of covariates (Figure 2). 

### 3.3. Primary Outcomes in Mathced Cohorts

**Figure 3:**
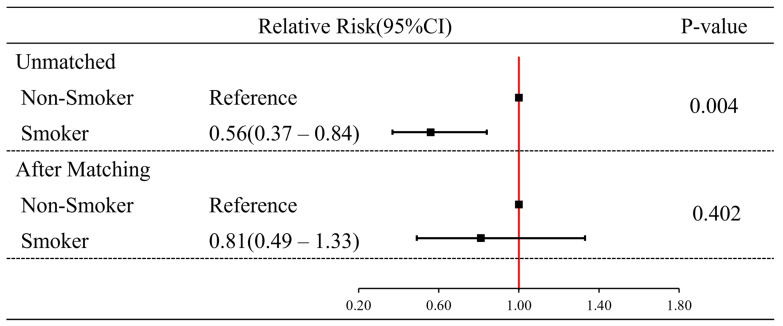
Comparison of relative risk for poor outcomes at 1 month between the non-smoking and smoking groups. CI: confidence interval.

Table 1 presents comparisons of neurocognitive outcomes at 1 month after CO exposure, stratified by current smoking status in the matched cohort. For the 317 matched pairs, neurocognitive outcomes did not show a statistically significant difference between the non-smoking and smoking groups (*p =* 0.461). In comparing non-smokers and smokers before PSM, the difference in poor outcome rates between non-smoker and smoker groups was statistically significant (χ^2^ = 8.77, df = 1, *p =* 0.004). After PSM, the difference in poor outcome rates between non-smoker and smoker groups was not significantly different (χ^2^ = 0.71, df = 1, *p =* 0.461). In addition, in terms of comparison of relative risk between non-smokers and smokers before PSM, a relative risk for the incidence of poor outcome was approximately 0.56 times lower in smokers than in non-smokers [0.56 (95% CI: 0.38–0.84, *p =* 0.004)]. However, after PSM, although the relative risk for the incidence of poor outcome was approximately 0.81 times lower in smokers than in non-smokers, no statistically significant difference was observed [0.81 (95% CI: 0.49–1.33, *p =* 0.402)] (Figure 3).

### 3.4. Additional Analysis for Neurocognitive Outcomes at 6 Months After Acute CO Poisoning

In an additional analysis evaluating 6-month neurocognitive outcomes, we found no statistically significant association between smoking status and poor outcomes, both before and after propensity score matching. In the unmatched cohort, the relative risk for smokers was 0.76 (95% CI: 0.50–1.17, *p* = 0.253). After matching, the relative risk increased to 1.21 (95% CI: 0.67–2.18, *p* = 0.632), although this increase was not statistically significant. These findings are consistent with our 1-month results, suggesting that smoking status does not significantly influence neurocognitive outcomes after acute CO poisoning, even over a longer follow-up period. Detailed results are provided in Supplementary Materials (Figure S1 and Table S2).

## 4. Discussion

Following propensity score matching for severity-related variables of CO poisoning, smoking status did not show a significant difference in terms of poor outcomes. Furthermore, the relative risk for neurocognitive outcomes did not demonstrate statistical significance. Our findings differ from those of Nah et al., who argued that a history of smoking may inversely correlate with the onset of delayed neurological sequelae (DNS) in cases of acute CO poisoning [39]. To explain the favorable prognosis associated with smoking, they posited the “neuroprotective effect by smoking paradox”. Numerous investigations have highlighted the so-called “smoker’s paradox”, in which individuals who smoke appear to have more favorable outcomes in cardio-cerebrovascular conditions as compared to non-smokers [23,40,41,42,43,44]. However, simultaneously it remains crucial to highlight that smoking use is a significant risk factor for cardio-cerebrovascular diseases. Smoking has been shown to be directly responsible for up to a quarter of all stroke cases, with the degree of risk being commensurate with the amount smoked and the risk of stroke decreased significantly after smoking cessation [20,45,46]. This correlation is not exclusive to stroke but extends to other cardiovascular conditions like myocardial infarction [47,48,49].

Recent studies have posited various theories to explain the so-called “smoker’s paradox”, wherein smoking appears to have certain beneficial outcomes [50,51]. One theory suggests that a potential bias could be the pre-selection of survivors among smokers admitted to hospitals [44]. Furthermore, the seemingly improved outcomes among smokers may not be directly attributable to smoking but rather an artifact of variables such as younger age, similar to outcomes before performing PSM in this study [52]. In this study, the smoking group was significantly younger than the non-smoking group in terms of age before PSM. Therefore, it is important to adjust confounding factors in terms of evaluating the effect of current smoking on neurological outcome [50,53]. We reduced this bias by adjusting variables that were significantly different between the two groups using PSM.

Our study presents several distinctive features compared to prior investigations exploring the association between CO poisoning and smoking [39]. Firstly, our study encompasses a more extensive patient population. Secondly, to address potential confounders related to the severity of CO poisoning, we applied a more robust statistical methodology by employing 1:1 propensity score matching. This statistical approach effectively addresses the inevitable disparities in demographic data between the two aforementioned groups, allowing us to isolate the specific effects of smoking. Thirdly, we assessed all patients’ neurocognitive outcomes at the 1-month with CO poisoning, although studies by Nah et al. [39] included relatively small sample sizes and only focused on estimating DNS. Lastly, we employed the GDS to assess patients’ neurocognitive outcomes. GDS was a scale typically developed in evaluating dementia patients [28], reflecting their actual social functioning. The GDS score has the advantage of recognizing neurocognitive function such as memory and concentration and activities of daily life through an interview, making it more objectively and easily to evaluate neurocognitive sequelae.

There are few limitations in this study. First, this study was a retrospective, observational study. Despite our analytical efforts to minimize bias, the possibility of hidden biases cannot be completely ruled out. Second, the study was conducted in a single center. As all patients were from a single geographical area, the generalizability to other regions may be limited. Therefore, to obtain more reliable results, another multicenter study should be conducted in the future. Third, our study included only Korean patients, and there may be variations in results across different racial and geographical groups. Forth, there is a relatively high proportion of patients who were excluded from the study. Among them, 398 patients failed the 1-month follow-up, making up the largest proportion despite efforts to reduce follow-up loss. These patients may have a poor prognosis that prevented them from participating in follow-up, which may have led to selection bias in the results. Fifth, despite our best efforts to achieve maximal similarity between the two groups through PSM, complete matching was not achieved for certain variables, such as patients’ psychiatric history and alcohol co-ingestion. This limitation is attributed to the difference in the pre-matching sample size between the two groups. It is essential to consider the potential impact of these variables on patients’ neurocognitive outcomes. Sixth, in the criteria for diagnosing CO poisoning, the COHb level was set differently from 5% for non-smokers to 10% for smokers. This is a reliable diagnostic standard used in several previous papers. However, since our study aims to compare prognosis between two groups according to smoking status, it may be necessary to consider whether the study was conducted on the exact same disease group. Seventh, during the PSM procedure, we also included HBO_2_ treatment status. However, our criteria for implementing HBO_2_ include patients initially presenting with neurological symptoms and COHb exceeding 25%. As a result, the variable—the usage of HBO_2_—might not be entirely independent. Since there was no notable change in the proportion of patients who received HBO_2_ before and after PSM, this effect is thought to be minimal, but the possibility of such biases must be considered. Eighth, when assessing smoking status, we categorized patients based on their current smoking status. Therefore, factors such as past smoking history and the duration of smoking were not reflected. Lastly, smoking status in our study was classified only as a binary variable (smoker vs. non-smoker) without consideration of smoking intensity, such as the number of cigarettes smoked per day or pack-years. This oversimplification may have masked potential dose-dependent associations between smoking exposure and neurocognitive outcomes after carbon monoxide poisoning.

## 5. Conclusions

In this study, current smoking status was not a predictor for neurological outcome.

## Figures and Tables

**Table 1:** Comparison of baseline characteristics in before and after propensity score matching.

	Unmatched			Matched (1:1)			
	Non-Smoking (*n* = 709; 61.7%)	Smoking (*n* = 441; 38.3%)	*p*-Value	Non-Smoking (*n* = 317)	Smoking (*n* = 317)	SMD	*p*-Value
Age	50 (38–64)	41 (32–54)	<0.001	46 (34–58)	43 (33–56)	−0.120	0.076
Sex			<0.001			0.044	0.504
Woman	383 (54.0)	60 (13.6)		49 (15.5)	55 (17.4)		
Man	326 (46.0)	381 (86.4)		268 (84.5)	262 (82.6)		
Intentionality	166 (23.4)	240 (54.4)	<0.001	123 (38.8)	140 (44.2)	−0.115	0.086
CO source			0.538			0.019	0.142
Charcoal	510 (71.9)	330 (74.8)		234 (73.8)	229 (72.2)		
Oil and gas	97 (13.7)	56 (12.7)		43 (13.6)	49 (15.5)		
Fire	102 (14.4)	55 (12.5)		40 (12.6)	39 (12.3)		
Drug co-ingestion	44 (6.2)	45 (10.2)	0.014	30 (9.5)	29 (9.1)	−0.011	1.000
GCS score	15 (12–15)	15 (12–15)	0.073	15 (12–15)	15 (12–15)	−0.017	0.817
Co-morbidities							
Diabetes mellitus	82 (11.6)	40 (9.1)	0.182	33 (10.4)	30 (9.5)	0.031	0.788
Hypertension	156 (22.0)	59 (13.4)	<0.001	53 (16.7)	50 (15.8)	0.025	0.826
Cardiovascular disease	31 (4.4)	13 (2.9)	0.221	13 (4.1)	11 (3.5)	−0.032	0.839
Psychiatric disease	61 (8.6)	76 (17.2)	<0.001	35 (11.0)	59 (18.6)	0.222	0.011
Alcohol co-ingestion	25 (3.5)	60 (13.6)	<0.001	20 (6.3)	36 (11.4)	0.177	0.029
Symptoms and signs at the ED							
Loss of consciousness	349 (49.2)	247 (56.0)	0.025	167 (52.7)	173 (54.6)	−0.038	0.673
Shock	24 (3.4)	10 (2.3)	0.277	9 (2.8)	9 (2.8)	0	1.000
Seizure	11 (1.6)	4 (0.9)	0.349	3 (0.9)	3 (0.9)	0	1.000
Use of HBO_2_ therapy	586 (82.7)	364 (82.5)	0.961	267 (84.2)	262 (82.6)	0.046	0.635
Number of HBO_2_ therapy sessions	1 (1–2)	1 (1–2)	0.524	1 (1–2)	1 (1–2)	−0.009	0.911
CO exposure time (hours)	4 (1–8)	3 (1–8)	0.007	3 (1–8)	3 (1–8)	0.002	0.974
Laboratory findings							
CO-Hb (%)	16.1 (5.7–28.7)	16.1 (6.6–30)	0.324	19.4 (6.5–29.6)	16.3(6.7–30)	−0.043	0.605
Bicarbonate (mmol/L)	21.8 (19.4–23.8)	21.6 (19.4–23.3)	0.099	21.5 (18.7–23.6)	21.6 (18.8–23.4)	−0.069	0.388
Lactate (mmol/L)	2.0 (1.3–3.1)	2.1 (1.4–3.5)	0.014	2.2 (1.38–3.42)	2.1 (1.37–3.69)	0.106	0.207
Creatinine (mg/dL)	0.78 (0.625–0.96)	0.87 (0.71–1.03)	<0.001	0.90 (0.74–1.06)	0.89 (0.72–1.1)	0	0.996
Creatine kinase (U/L)	130 (84–280)	143 (96.5–256)	0.027	147 (103–293)	151 (99–311)	0.024	0.745
Troponin I (ng/mL)	15 (12.67–146)	15 (9–128)	0.051	15 (11–115)	15 (8–187)	−0.009	0.852
Intubation			<0.001				
Yes	51 (7.2)	29 (6.6)		21 (6.62)	23 (7.26)	0.104	0.600
Outcome	86 (12.1)	30 (6.8)	0.004	31 (9.8)	25 (7.9)	−0.064	0.461

Data are expressed as frequency (percentage), mean ± standard deviation, and median (interquartile range). SMD: standardized mean difference, CO: carbon monoxide, GCS: Glasgow coma scale, ED: emergency department; HBO_2_: hyperbaric oxygen, CO-Hb: carboxyhemoglobin.

## Data Availability

The datasets generated and/or analyzed during the current study are not publicly available but are available from the corresponding author upon reasonable request.

## Supplementary Materials

The following supporting information can be downloaded at: https://www.mdpi.com/article/10.3390/jcm14072497/s1, Table S1: Global deterioration scale. Table S2: Baseline characteristics of the propensity score matched and unmatched cohorts at 6 months after acute CO poisoning. Figure S1: Comparison of relative risk of poor outcomes at 6 months after acute CO poisoning between the non-smoking and smoking groups. CI: confidence interval; CO: carbon monoxide.

## Abbreviations

The following abbreviations are used in this manuscript:

SMD	Standardized Mean Difference
CO	Carbon Monoxide
GCS	Glasgow Coma Scale
ED	Emergency Department
HBO_2_	Hyperbaric Oxygen
CO-Hb	Carboxyhemoglobin
PSM	Propensity Score Matching
CI	Confidence Interval
