# Supplementary material for: Effects of Smoking on Neurocognitive Outcomes in Patients with Carbon Monoxide Poisoning
Source: J Clin Med. 2025 Apr 6;14(7):2497. doi: 10.3390/jcm14072497 (PMC11989691; doi:10.3390/jcm14072497)
Supplement: Supplementary file 1 [file jcm-14-02497-s001.zip › jcm-3546021-supplementary.pdf]

Supplementary Materials

Table S1. Global deterioration scale.

Stage	Cognitive dysfunction	Clinical characteristics
1	No cognitive decline	*Patients appear clinically normal*<br>No complaints of memory deficits.<br>No evident memory deficit in the clinical interview.
2	Very mild cognitive decline	*Patients complain of memory deficits*<br>Most frequently, patients<br>(a) Forget where they have placed familiar objects<br>(b) Forget the name of someone they formerly knew well.<br>No objective evidence of memory deficit in clinical interviews.<br>No objective deficits in employment or social situations.<br>Patients display appropriate concern about their symptoms.
3	Mild cognitive decline	*Earliest clear-cut deficits*<br>Objective evidence of memory deficit was obtained only through an intensive interview conducted by a trained geriatric psychiatrist. Concentration deficit may be evident in clinical testing.<br>Patients may demonstrate a reduced ability to<br>(a) Remember names upon introduction to new people<br>(b) Retain information after reading a passage from a book<br>Decreased performance manifests in demanding employment and social situations. Examples may include:<br>(a) Coworkers becoming aware of the patient's relatively poor performance<br>(b) Difficulties in finding words and names becoming evident to intimate acquaintances<br>(c) Losing or misplacing objects of value<br>(d) Getting lost when traveling to unfamiliar locations<br>The subtlety of the clinical symptoms may be exacerbated by denial that often manifests in these patients. Mild-to-moderate anxiety also accompanies the symptoms, typically when the patients are forced to cope with challenging employment and social demands that they find they can no longer negotiate.
4	Moderate cognitive decline	*Clear-cut deficits on careful clinical interview*<br>Deficits manifest in many areas, such as<br>(a) Concentration deficit elicited in serial subtractions<br>(b) Decreased knowledge of current events and recent life events<br>(c) A deficit in the memory of their personal history upon careful questioning<br>(d) Decreased ability to travel alone and manage finances<br>Patients can no longer perform complex tasks accurately and efficiently. However, certain abilities remain preserved, such as<br>(a) Orientation to time and people<br>(b) Familiar persons and faces can be distinguished from strangers

		(c) Ability to travel to familiar locations<br>Denial is often the dominant defense mechanism. The evident decline in the patients' intellectual and cognitive capacities is too overwhelming a loss for full conscious acceptance and recognition. A flattening of efficacy and withdrawal from previously challenging situations are observed.
5	Moderately severe cognitive decline	*Patients can no longer survive without some assistance*<br>During interviews, patients are unable to recall a major relevant aspect of their current lives. Examples include:<br>(a) Difficulty recalling their address or telephone number, names of close family members, such as grandchildren, or the name of the high school or university from which they graduated<br>(b) Some disorientation with time (date, day of the week, season) or location<br>(c) Well-educated patients may have difficulty counting backward from 40 by fours or from 20 by twos.<br>Patients retain the knowledge of many major facts regarding themselves and others. They invariably know their own names and generally know their spouses' and children's names. They require no assistance with toileting and eating but may have some difficulty choosing the proper clothing to wear and may occasionally clothe themselves improperly (e.g., putting their shoes on the wrong feet).
6	Severe cognitive decline	*Patients may occasionally forget the name of their spouse, on whom they depend entirely for survival*<br>Patients are largely unaware of all recent events and experiences in their lives.<br>They retain some knowledge of their past, but this knowledge is very uncertain. They are generally unaware of their surroundings, the year, or the season and may have difficulty counting backward, and sometimes forward, from 10. Patients require substantial assistance with activities of daily living. These symptoms are quite variable and include:<br>(a) Delusional behavior (e.g., patients may accuse their spouse of being an impostor, may talk to imaginary figures in the environment, or their own reflection in the mirror)<br>(b) Obsessive symptoms (e.g., continual repetition of simple cleaning activities)<br>(c) Anxiety symptoms, agitation, and previously nonexistent violent behavior<br>(d) Cognitive abulia (i.e., loss of willpower because they cannot carry a thought long enough to determine a purposeful course of action).
7	Very severe cognitive decline	*All verbal abilities are lost*<br>Frequently, there is no speech ability at all; only grunting remains.<br>Patients have urinary incontinence and require assistance with toileting and eating. They lose psychomotor skills, such as the ability to walk. The brain appears unable to tell the body what

	to do. Generalized cortical and focal neurologic signs and symptoms are frequently present.

Table S2. Baseline characteristics of the propensity score matched and unmatched cohorts at 6 months after acute CO poisoning

	Unmatched			Matched (1:1)			
	**Non-smoking (n=675; 62.4%)**	**Smoking (n=406; 37.6%)**	***p*-value**	**Non-smoking (n=327)**	**Smoking (n=327)**	**SMD**	***p*-value**
Age	50 (38-64)	42 (33-54)	<0.001	44.0 (34-57)	42 (33-55)	0.101	0.266
Sex			<0.001			0.223	0.059
Woman	363 (53.8)	56 (13.8)		23 (7.0)	38 (11.6)		
Man	312 (46.2)	350 (86.2)		304 (93.0)	289 (88.4)		
Intentionality	153 (22.7)	216 (53.2)	<0.001	178 (54.4)	194 (59.3)	0.140	0.236
CO source			0.522			0.162	0.166
Charcoal	476 (70.5)	299 (73.6)		273 (83.5)	267 (81.7)		
Oil and gas	97 (14.4)	54 (13.3)		29 (8.9)	42 (12.8)		
Fire	102 (15.1)	53 (13.1)		25 (7.6)	18 (5.5)		
Drug co-ingestion	40 (5.9)	35 (8.6)	0.108	36 (11.0)	32 (9.8)	0.057	0.701
GCS score	15 (12.0-15.0)	15 (12.0-15.0)	0.086	15 (12.0-15.0)	15 (12.0-15.0)	0.037	0.892
Co-morbidities							
Diabetes mellitus	77 (11.4)	38 (9.4)	0.310	42 (12.8)	33 (10.1)	0.122	0.326
Hypertension	148 (21.9)	55 (13.5)	0.001	57 (17.4)	48 (14.7)	0.106	0.394
Cardiovascular disease	29 (4.3)	12 (3.0)	0.325	12 (3.7)	9 (2.8)	0.074	0.658
Psychiatric disease	57 (8.4)	65 (16.0)	<0.001	64 (19.6)	56 (17.1)	0.090	0.480
Alcohol co-ingestion	21 (3.1)	54 (13.3)	<0.001	18 (5.5)	49 (15.0)	0.442	<0.001
Symptoms and signs at the ED							
Loss of consciousness	325 (48.1)	223 (54.9)	0.033	221 (67.6)	200 (61.2)	0.190	0.102
Shock	15 (2.2)	7 (1.7)	0.661	5 (1.5)	7 (2.1)	0.064	0.772

Seizure	10 (1.5)	4 (1.0)	0.587	3 (0.9)	4 (1.2)	0.042	1.000
Use of HBO_2 therapy	560 (83.0)	335 (82.5)	0.868	327 (100.0)	327 (100.0)	0	1.000
Number of HBO_2 therapy sessions	1 (1-2)	1 (1-2)	0.979	1 (1-2)	1 (1-2)	0.043	0.497
CO exposure time (hours)	4 (1-8)	3 (1-8)	0.007	2.9 (1.0-8.0)	3 (1.0-8.0)	0.036	0.334
Laboratory findings							
CO-Hb (%)	16.1 (5.8-29.0)	16.0 (6.7-30.0)	0.429	21.0 (8.0-31.0)	18.6 (8.0-31.0)	0.008	0.860
Bicarbonate (mmol/L)	21.8 (19.5-24.0)	21.6 (19.3-23.0)	0.053	21.2 (19.4-24.0)	21.5 (18.8-23.0)	0.145	0.315
Lactate (mmol/L)	2.0 (1.3-3.0)	2.1 (1.0-4.4)	0.011	2.5 (1.5-3.0)	2.2 (1.4-4.0)	0.043	0.386
Creatinine (mg/dL)	0.8 (0.6-1.0)	0.9 (0.7-1.0)	<0.001	1.0 (0.8-1.0)	0.9 (0.7-1.0)	0.154	0.001
Creatine kinase (U/L)	130.0 (84.5-275.0)	142.0 (96.0-252)	0.049	138 (104.0-340.0)	143 (97.0-266.0)	0.121	0.464
Troponin I (ng/mL)	15.0 (12.7-131)	15.0 (9.1-98)	0.086	16 (13.0-156.0)	15 (15.0-132.0)	0.108	0.212
Intubation			0.269				
Yes	45 (6.7)	23 (5.7)		15 (4.6)	22 (6.7)	0.183	0.245
Outcome	61 (9.0)	28 (6.9)	0.253	19 (5.8)	23 (7.0)	0.071	0.633

Data are expressed as frequency (percentage), mean ± standard deviation, and median (interquartile range). SMD: standardized mean difference, CO: carbon monoxide, GCS: Glasgow coma scale, ED: emergency department; HBO2: hyperbaric oxygen, CO-Hb: carboxyhemoglobin.

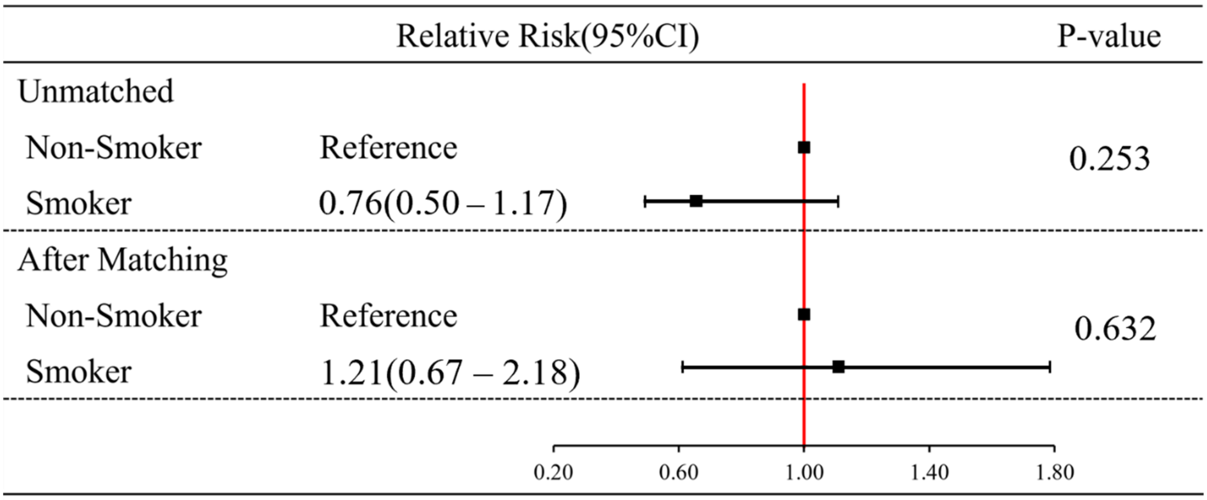

Figure S1. Comparison of relative risk of poor outcomes at 6 months after acute CO poisoning between the non-smoking and smoking groups. CI: confidence interval; CO: carbon monoxide
